# Data representing two separate LC-MS methods for detection and quantification of water-soluble and fat-soluble vitamins in tears and blood serum

**DOI:** 10.1016/j.dib.2017.02.033

**Published:** 2017-02-16

**Authors:** Maryam Khaksari, Lynn R. Mazzoleni, Chunhai Ruan, Robert T. Kennedy, Adrienne R. Minerick

**Affiliations:** a Department of Chemical Engineering, Michigan Technological University, 1400 Townsend Drive, Houghton, MI 49931, USA; b Department of Chemistry, Michigan Technological University, 1400 Townsend Drive, Houghton, MI 49931, USA; c Metabolomics Core, BRCF, University of Michigan, 500 South State Street, Ann Arbor, MI 48109, USA; d Department of Chemistry, University of Michigan, 500 South State Street, Ann Arbor, MI, 48109, USA

**Keywords:** LC-MS method, Tears, Blood serum, Water-soluble vitamin, Fat-soluble vitamin, Infant, Parent

## Abstract

Two separate liquid chromatography (LC)-mass spectrometry (MS) methods were developed for determination and quantification of water-soluble and fat-soluble vitamins in human tear and blood serum samples. The water-soluble vitamin method was originally developed to detect vitamins B_1_, B_2_, B_3_ (nicotinamide), B_5_, B_6_ (pyridoxine), B_7_, B_9_ and B_12_ while the fat-soluble vitamin method detected vitamins A, D_3_, 25(OH)D_3,_ E and K_1_. These methods were then validated with tear and blood serum samples. In this data in brief article, we provide details on the two LC-MS methods development, methods sensitivity, as well as precision and accuracy for determination of vitamins in human tears and blood serum. These methods were then used to determine the vitamin concentrations in infant and parent samples under a clinical study which were reported in "Determination of Water-Soluble and Fat-Soluble Vitamins in Tears and Blood Serum of Infants and Parents by Liquid Chromatography/Mass Spectrometry DOI:10.1016/j.exer.2016.12.007 [1]". This article provides more details on comparison of vitamin concentrations in the samples with the ranges reported in the literature along with the medically accepted normal ranges. The details on concentrations below the limits of detection (LOD) and limits of quantification (LOQ) are also discussed. Vitamin concentrations were also compared and cross-correlated with clinical data and nutritional information. Significant differences and strongly correlated data were reported in [1]. This article provides comprehensive details on the data with slight differences or slight correlations.

**Specifications Table**

**Table:** 

Subject area	Analytical Chemistry
More specific subject area	Liquid Chromatography, Mass Spectrometry, Vitamin Analysis
Type of data	Table, Figure
How data was acquired	Accela LC and LCQ Fleet mass spectrometer with electrospray ionization (ESI)
Data format	Raw and analyzed data using xcalibur software (version C)
Experimental factors	Tear and blood serum samples were prepared under the procedure described in [1].
Experimental features	Mobile phases A and B for water-soluble vitamin method were 0.1% FA in water and 0.1% FA in ACN with gradient of 0 min, 100% A; 7 min, 100% A; 12 min, 50% A; 18 min, 5% A and flow rate of 0.2 mL/min. Mobile phases A and B for fat-soluble vitamin method were 9:1 (v/v) ACN/water and 100% MeOH, both containing 5 mM ammonium formate with gradient of 0 min, 100% A; 1 min, 100% A; 6 min, 0% A; 25 min, 0% A and flow rate of 0.2 mL/min. The analysis in both methods was done over three time segments with analyte optimized ESI-MS parameters in each segment. Using MS/MS, a suitable fragment ion for each vitamin was isolated under selected ion monitoring (SIM) and selected reaction monitoring (SRM) modes and the peak area of the fragment ion was used for quantification.
Data source location	Not applicable
Data accessibility	Data is with this article

**Value of the data**

• The two separate LC-MS methods described herein can be used for simultaneous detection and quantification of eight water-soluble vitamins in under 16 min and simultaneous detection and quantification of five fat-soluble vitamins in under 25 min.
• These two methods can be applied to analysis of tears and blood serum sample vitamin levels or any other types of samples with appropriate sample preparation adjustments.
• The use of internal standards (IS) simplifies the sample preparation and can compensate for matrix effects or compound losses during sample preparation.
• The sensitivity of the proposed methods is sufficient to be used for detection and quantification of vitamin concentrations in biofluids for vitamin deficiency diagnosis or food quality.

## 1 Data

### 1.1 Detection of water-soluble vitamins with developed LC-MS method

**Fig. 1:**
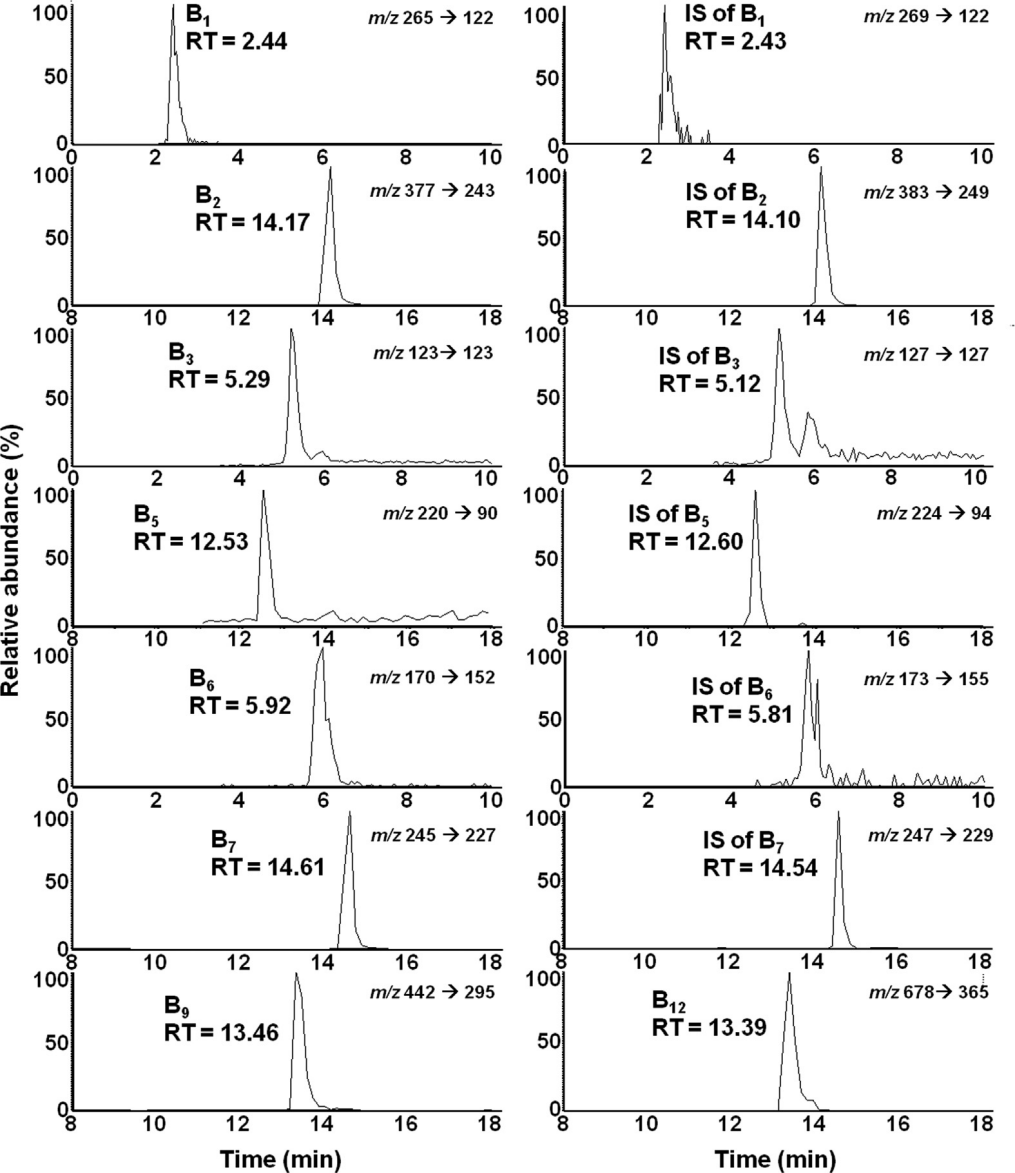
Chromatograms of the water-soluble vitamins generated using a standard solution under MS/MS analysis. Plots illustrate a 10-min window around each vitamin peak. The peaks represent the fragment ions generated using the selected reaction monitoring (SRM) mode. Analyte retention times are also shown near each of the analyte peaks. For vitamin B_9_ and B_12_ the area of the vitamin B_2_ internal standard (IS) was used. The co-eluting peaks of B_9_ and B_12_ are distinguished via MS/MS analysis.

**Fig. 2:**
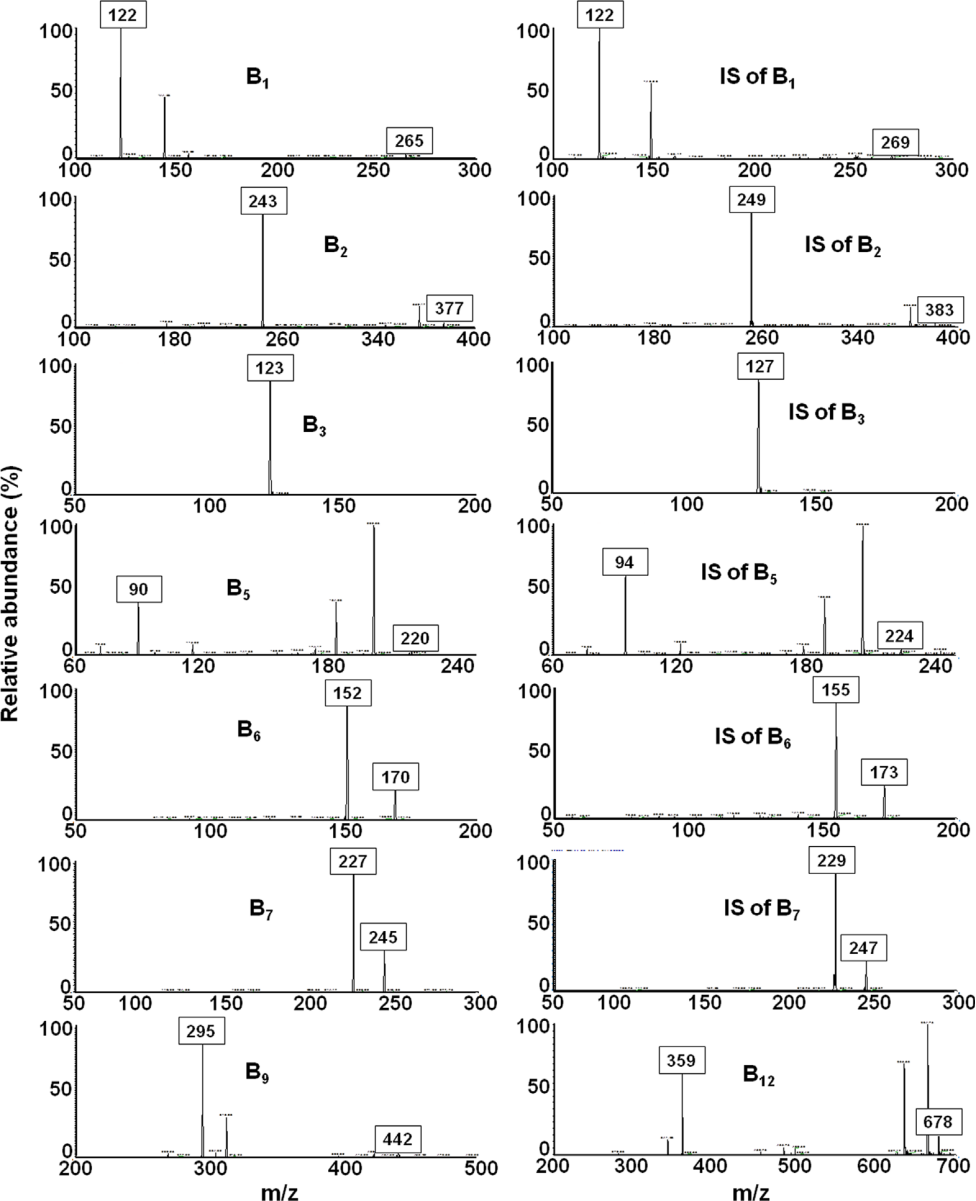
MS/MS spectra of the water-soluble vitamins and their stable isotope internal standards. Precursor ions of vitamins and the fragment ions used for quantification are labeled on the spectra.

Water-soluble vitamins and their internal standards were retained in the chromatography column < 16 min with 2.48±0.07, 14.11±0.08, 5.33±0.04, 12.60±0.07, 6.02±0.10, 14.55±0.08, 13.39±0.07, 13.39±0.01 min retention times for B_1_, B_2_, B_3_, B_5_, B_6_, B_7_, B_9_, B_12_, respectively, and 2.51±0.07, 14.11±0.02, 5.15±0.04, 12.60±0.00, 5.91±0.10, 14.50±0.07 min for B_1_ IS, B_2_ IS, B_3_ IS, B_5_ IS, B_6_ IS and B_7_ IS, respectively. The chromatograms and the spectra achieved by a standard solution of water-soluble vitamins are shown in Fig. 1, Fig. 2, respectively.

**Fig. 3:**
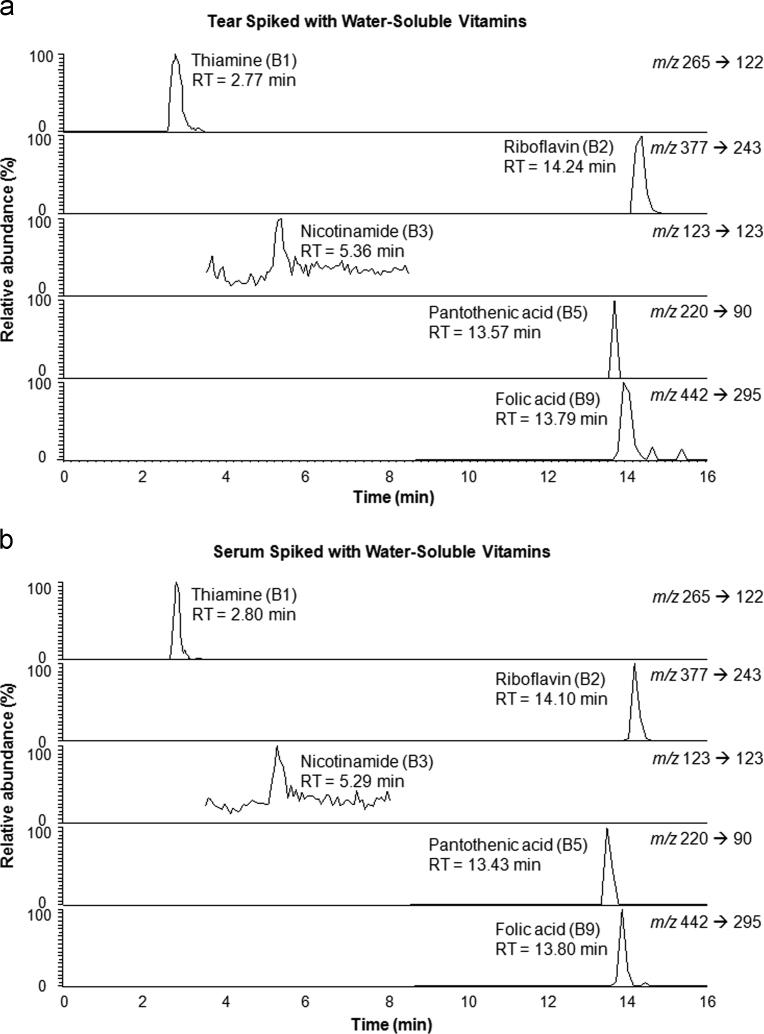
Chromatograms of water-soluble vitamins in: a) tear and b) serum both spiked with 0.5 μM water-soluble vitamin standard solutions. Chromatograms were generated by reverse-phase high-pressure liquid chromatography (LC) with the positive-ion mode electrospray ionization mass spectrometry (ESI-MS) with MS/MS analysis. Vitamins B_1_ (thiamine), B_2_ (riboflavin), B_3_ (nicotinamide), B_5_ (pantothenic acid) and B_9_ (folic acid) were detected in both tear and serum and were eluted in under 16 min.

Tear and blood samples were prepared under the water-soluble vitamin procedure described in [1] and analyzed by the water-soluble vitamin LC-MS method. Vitamins B_1_, B_2_, B_3_, B_5_ and B_9_ were detected in both tear and blood serum while vitamins B_6_, B_7_ and B_12_ were not detected. Fig. 3 shows the chromatograms of water-soluble vitamins generated using SRM mode in a tear (Fig. 3a) and a serum (Fig. 3b) sample both spiked with 0.5 μM water-soluble vitamin standard solutions.

### 1.2 Detection of fat-soluble vitamins with developed LC-MS method

**Fig. 4:**
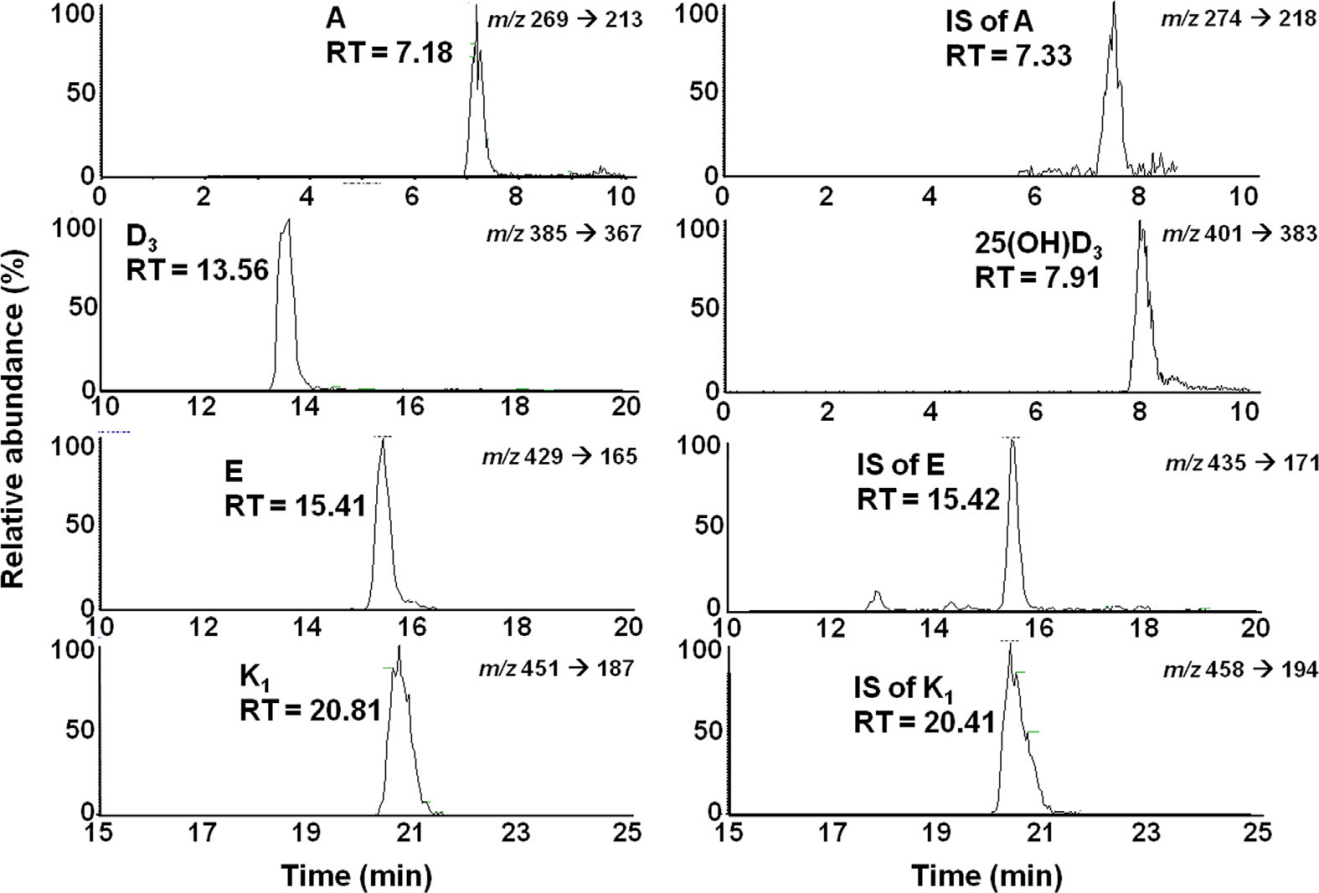
Chromatograms of the fat-soluble vitamins generated by a standard solution using MS/MS analysis. The plots illustrate a 10-minute window around each vitamin peak. The peaks represent the fragment ions generated using the selected reaction monitoring (SRM) mode. Retention times are shown for each of the analyte peaks. For vitamins D_3_ and 25(OH)D_3_ the area of the vitamin E internal standard (IS) was used.

**Fig. 5:**
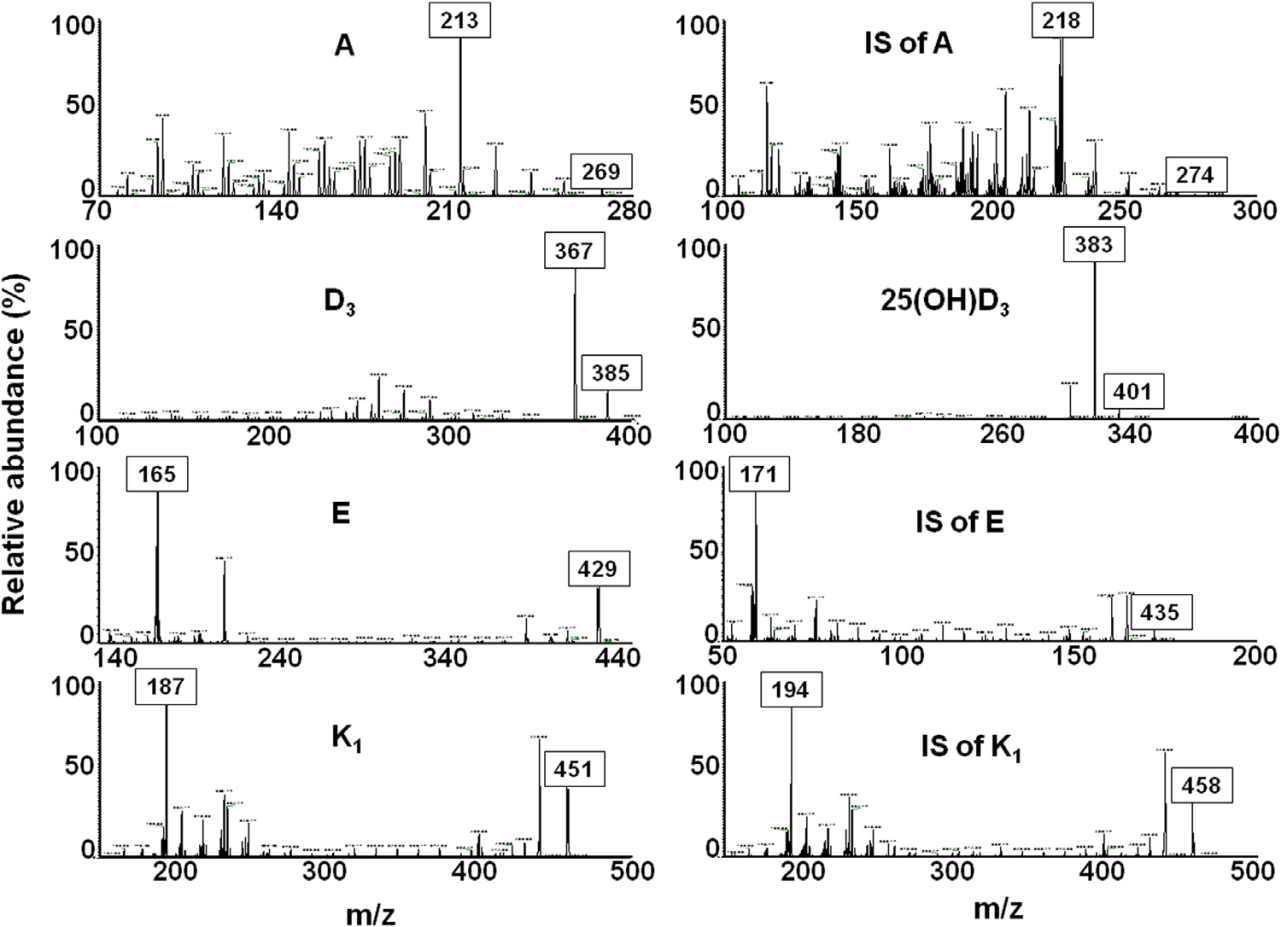
MS/MS spectra of the fat-soluble vitamins and their stable isotope internal standards. Precursor ions of vitamins and the fragment ions used for quantification are labeled on the spectra.

Fat-soluble vitamins and their ISs were retained in the chromatography column within 25 min with 7.19±0.05, 7.91, 13.53±0.03, 15.37±0.04, 20.74±0.07 min retention times for A, D_3_, 25(OH)D_3_, E, K_1_ and 7.33±0.04, 15.46±0.04 and 20.47±0.06 min for A IS, E IS and K_1_ IS, respectively. The chromatograms and spectra achieved by a standard solution of fat-soluble vitamins generated under SRM mode are shown in Fig. 4, Fig. 5, respectively.

**Fig. 6:**
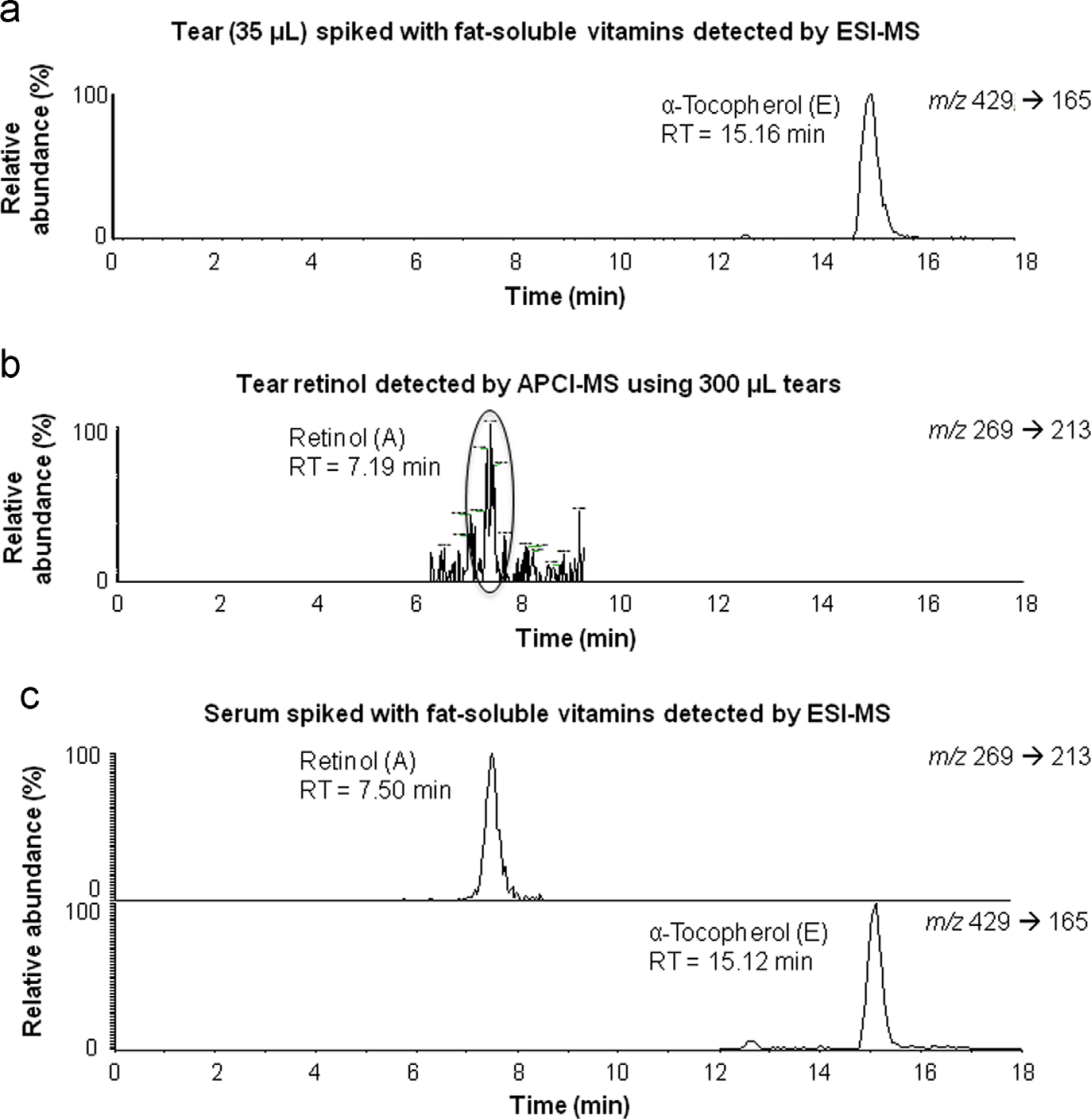
Chromatograms of the fat-soluble vitamins in: a) tear spiked with 0.5 μM vitamin E detected by ESI-MS, b) tear prepared via Speek at al. [2] method and detected with APCI-MS, c) serum spiked with 1 μM vitamin A and 10 μM vitamin E detected by ESI-MS. Peaks were generated by reverse-phase high-pressure liquid chromatography with the positive-ion mode electrospray using the selected reaction monitoring (SRM) mode. Vitamins eluted in under 18 minutes.

Tear and blood samples were prepared under the fat-soluble vitamin procedure described in [1] and analyzed by the fat-soluble vitamin LC-MS method. Vitamin E was detected in both tear and blood serum while vitamin A was only detected in serum. Other fat-soluble vitamins (D_3_, 25(OH)D_3_ and K) were not detected in tears and serum. Chromatograms of a tear and a serum sample spiked with fat-soluble vitamin standard solutions detected with ESI probe are shown in Figs. 6a and 6c, respectively. The absence of several fat-soluble vitamins in tears suggests low vitamin concentrations or MS ionization interferences. Ionization of fat-soluble vitamins with ESI is difficult because they do not have functional groups in their structure to easily accept or donate electron. We added ammonium formate in the fat-soluble vitamin mobile phases to enhance their ionization. However, the low concentration of vitamins in the biological samples made it such that the analytes were not detectable in the MS spectra. To examine the concentration and ionization attributes, 300 μL tear samples were collected with glass capillaries, prepared via the Speek et al. [2] method, and then analyzed with ESI and also with an atmospheric pressure chemical ionization (APCI) probe. As shown in Fig. 6b, positive APCI can discern vitamin A while positive ESI cannot. This suggests that gasification prior to ionization is superior, because the APCI behavior is linear at low concentrations, while the analyte has a detrimental nonlinearity in ESI at low concentrations [3]. The presence of 25(OH)D in tears was verified using enzyme-linked immunosorbent assay (ELISA) with LOD of 1.6 ng/mL (data not shown).

### 1.3 LOD, LOQ, precision and accuracy

The calibration solutions were prepared following the method described in [1]. Separate calibration curves were generated for tears and serum. LOD and LOQ were determined by the replicate injections (n=7) of a low-level sample (tears or blood serum) and calculating the signal standard deviations. The LOD and LOQ were defined as 3 and 10 times the standard deviations divided by the slope of the linear calibration curve for each vitamin [4]. Table 1 reports calibration equations, ranges of linearities, LOD and LOQ for both tears and blood serum. For most water-soluble vitamins, tear LOD were higher than for blood serum. Since the instrument conditions were constant, the LOD differences noted between serum and tear samples could be due to the sample extraction method or matrix effects. However, due to the presence of ISs, matrix effects were deemed to not contribute to the differing LOD. Thus, the extraction methods provide a more likely explanation of the higher LOD values in tears than blood serum. For fat-soluble vitamins, vitamin E was detected at much higher concentrations in serum than in tears while the LOD in tears was lower than serum. Tears have lower lipid content and likely reduce the interference of undesired lipid compounds in the detection procedure.

Intra-day (n=6) and inter-day (n=7) precision and accuracy (Table 2) were determined by spiking serum and tears with three different concentrations of vitamin standard solutions. The relative standard deviations (RSD) were calculated for the precision and the extracted amounts were calculated for the recovery. Although vitamins B_5_ and B_9_ were detected in tear and serum, the recoveries of these vitamins were not sufficient likely due to the co-elution of B_5_ and B_9_ and/or potential tear interferences in ESI-MS. The plausibility of this explanation was supported by spiking tear extract with vitamins B_5_ and B_9_ (right before LC-MS injection); insufficient recovery was observed.

### 1.4 Vitamin concentrations in tears and blood serum

The two developed LC-MS methods were used to determine vitamin concentrations in tear and blood serum of 15 family pairs; each pair consisting of one four-month-old infant and one parent as reported in [1]. Here, the concentrations of vitamins determined in the infant/parent samples are compared against the ranges reported in the literature along with the medically accepted normal ranges. Any data falling below the LOD and LOQ are included here.

Vitamin B_1_ serum concentrations reported in [1] were in the range reported by other literature [5], [6]. The medically accepted normal range for blood serum B_1_ is reported to be 0.008–0.030 μM [7]. In 3 infants, serum B_1_ concentrations were just above (0.045–0.065 μM) the normal range. 5 infant and 5 parent tear, and 3 infant and 3 parent serum samples (not paired) did not exceed the LOD, nor the normal range.

Vitamin B_2_ serum concentrations reported in [1] were in the range reported by other literature [5], [8] and also the medically accepted normal range (0.003–0.050 μM [9]). 5 infants and 1 parent serum concentrations were above the normal range (0.060–0.15 μM). However, adult reference concentrations may not be appropriately accurate for infants.

Vitamin B_3_ serum concentrations reported in [1] found in infants and parents were higher than those in literature [5]; however, nitcotinamide overdoses do not cause vasodilatation or flushing and also do not decrease the lipid serum concentration. Concentrations were above the LOD except for one parent serum sample.

Vitamin B_5_ serum concentrations reported in [1] were in the range of other methods [10]. All 14 infant serum B_5_ were in the normal ranges (for children: 0.016–3.8 μM [9]), while 2 parent serum were just below (0.11 μM) and one parent serum (0.74 μM) was just above the normal range (for adults: 0.17–0.67 μM [9]). In one infant tear, one parent tear, and 3 parent serum samples, B_5_ peaks below the LOD were observed; they were classified as undetected in [1].

Vitamin B_9_ concentrations reported in [1] were below the LOD in 5 infant and 3 parent tear samples, and 2 infant and 3 parent serum samples. Serum concentrations we obtained were higher than those reported in other literature [11] or laboratory normal ranges (0.011–0.036 μM [12]). However in another reference, values below 0.091 μM are reported as normal [13].

Serum vitamin A concentrations were reported in [1]. One infant was below LOD and one infant (0.39 μM) was just below the normal range. In 3 parent serum samples, vitamin A concentrations (4.1– 6.1 μM) were above the normal range [9] and two parent samples were below the normal range.

Vitamin E serum concentrations reported in [1] were consistent with other literature [14], [15]. According to the clinical values [9], 3 infant (5.4–8.0 μM) and 4 parent serum (3.5 and 8.1 μM) samples were below the normal ranges.

### 1.5 Comparison of vitamin concentrations with clinical data and nutritional information

For the correlations study, Pearson product-moment correlation coefficient, C, was used [16]:

$$C_{a,b}=\;\frac{\sum (a-\bar{a})(b-\bar{b})}{\sqrt{\sum {(a-\bar{a})}^{2}\sum {(b-\bar{b})}^{2}}}$$

where a and b represent the data sets being compared and $\bar{a}$ and $\bar{b}$ are the mean values of data set a and b. The calculated C values are normalized in the formula to range from −1 to 1 with positive numbers showing positive correlations (i.e. if one data set increases, the second increases as well) and negative numbers showing negatively correlated data (one data set increases, the second decreases or vice versa). Statistical analyses were done according to [1] and are detailed here.

#### 1.5.1 Gender

Slight differences were noted for some vitamin concentrations by gender. Vitamin B_3_ concentrations were slightly greater in the tear and serum of male infants (p=0.12 for both tears and serum). Slightly higher vitamin A concentrations in female serum than in male serum (p=0.12) were achieved. For other vitamins in both sample types, no significant difference was found by infant gender.

#### 1.5.2 Age

Infants were 130±15 days old. Slight positive correlations with the infant age were observed for tear B_1_ concentrations (C=0.32) and serum B_1_ concentrations (C=0.32). The infant age was also slightly correlated to the serum B_2_ concentrations (C=0.30). Age correlations with vitamin E concentrations in tears and in serum were stronger (C=0.49 and 0.39, respectively). Due to the small age difference between infant participants, no strong correlations were obtained between vitamin concentrations and infant age. The only data provided in the literature are for water-soluble vitamin B_7_ and there are no correlations with age [17].

#### 1.5.3 Weight

Infant weights were 6.9±0.9kg. The sample population was roughly centered at the 50th percentile: high = 8.4 kg at 95th percentile, low = 5.8kg at 5th percentile [18]. A slight positive correlation with weight was only observed for the serum B_1_ concentrations (C=0.38). Meanwhile, concentrations of vitamin E in tears and serum were lower in infants with higher weights (C=−0.46 and −0.30,respectively).

#### 1.5.4 Length

Infant lengths were 64±2 cm with the sample population roughly centered at the 50th percentile [18]. Positive correlations were observed between the infant length and their serum vitamin B_1_ (C=0.33) concentrations. Negative correlations existed between the infant length and concentrations of vitamin E in tears and serum (C=−0.45 and −0.53, respectively).

#### 1.5.5 Head circumference

Infant head circumferences were reported for 13 infants in the range of 42.0±1.1 cm. Tear concentrations of B_3_ tended to slightly increase as head circumference increased (C=0.31). Meanwhile, vitamin A concentrations and head circumference were strongly negatively correlated (C=−0.51).

#### 1.5.6 Race/ethnicity

11 of 15 infants were white, 2 Asian/Pacific islanders, 1 American-Indian and 1 multicultural. Parents included 2 Asian/Pacific Islanders. The population numbers were insufficient to draw meaningful conclusions based upon race or ethnicity.

#### 1.5.7 Apgar scores

Apgar scores are a measure of a newborn׳s overall physical condition. Five factors are used to define this number: **A**ctivity (muscle tone), **P**ulse (heart rate), **G**rimace (reflex response), **A**ppearance (skin color), and **R**espiration (breathing). Each factor is scored in a scale of 0, 1 or 2 with 2 being the best score. The Apgar scores of 10 indicate a baby with the best conditions. The 1 min and 5 min Apgar scores were recorded for infants at birth. Six infants had 1 min Apgar scores of 9, 7 infants had 8 and 1 infant had 2. The Apgar score of 5 min was reported to be 9 for 13 infants and 8 for 1 infant. For one of the infants, the Apgar score was not reported. In infants with 1 min Apgar scores of 9, the serum concentrations of B_2_ and A were slightly higher (p=0.082 and 0.14, respectively) and serum concentrations of E were slightly lower (p=0.17) than infants with 1 min Apgar scores of 8 and 2.

All infants had normal muscle reflexes and appearances and 4 infants were reported to have reflux digestive issues. The population numbers with and without reflux issues were insufficient to draw meaningful conclusions. No vitamin/reflux correlations are reported in the literature with the exception that stomach acid assists vitamin B_12_ absorption, so adults treated with acid-reflux drugs can incur vitamin B_12_ deficiencies [19].

## 2 Experimental design, materials and methods

### 2.1 Materials and chemicals

The purchased standard vitamins B_1_, B_2_, B_3_, B_5_, B_9_, A, E, their corresponding IS, and all the solvents used were similar to [1]. Vitamins B_6_, pyridoxine dihydrochloride (≥98%), B_7_, biotin (≥99%, TLC), B_12_, cyanocobalamin (analytical standard), D_3_, cholecalciferol (pharmaceutical secondary standard), 25-hydroxycholecalciferol (≥98%, HPLC), K_1_, phylloquinone (analytical standard) and IS of K_1_, K-[5,6,7,8-D_4_, 2-methyl-D_3_] were purchased from Sigma-Aldrich (St. Louis, MO, USA). Pyridoxine-[D_3_] hydrochloride and biotin-[D_2_] were purchased from Isosciences (Trevose, PA, USA).

### 2.2 Standard solutions and sample preparation

5 mM stock solutions for B_6_ was prepared in water and for B_7_ and B_12_ were prepared in DMSO. Stock solutions of vitamins D_3_, 25-hydroxycholecalciferol and K were 50, 5 and 25 mM in MeOH, respectively. 5 mM pyridoxine-[D_3_] hydrochloride was prepared in D_2_O while 25 mM biotin-[D_2_] and 2.5 mM K-[5,6,7,8-D_4_, 2-methyl-D_3_] were prepared in MeOH. For other vitamins, stock and working solutions were prepared as described in [1]. Tear and blood samples were prepared under the water-soluble and fat-soluble vitamin extraction procedures described in [1].

### 2.3 Water-soluble vitamin LC-MS method

The water-soluble vitamin LC-MS method was completed in 18 min over three time segments. Voltages were optimized over time and after instrument maintenance for each segment; capillary and tube lens voltages were in the range of 17–46 and 65–115 V, respectively. The spray voltage and capillary temperature for all vitamins were set to 4 kV and 275°C, respectively. Vitamins were all detected in positive ESI mode. Nitrogen was used as the nebulizing gas at flow rates of 10 (arbitrary units). In each segment, three scans were recorded: 1) full scan with the ranges reported in Table 3, Table 2) selected ion monitoring (SIM) scan for isolating the precursor ions, and 3) selected reaction monitoring (SRM) mode for isolating the fragment ions of vitamins for quantifications. The vitamin molecular weights, precursor ions, collision energies and fragment ions used for quantifications are reported in Table 3.

### 2.4 Fat-soluble vitamin LC-MS method

The fat-soluble vitamin LC-MS method was done in 25 min over three time segments. Capillary and tube lens voltages were optimized over time and after instrument maintenance and were in the range of 1–22 and 60–70 V, respectively. The spray voltage and capillary temperature for all vitamins were set to 4 kV and 275 °C, respectively. Vitamins were all detected in positive ESI mode. Nitrogen was used as the nebulizing gas at flow rates of 20 (arbitrary units). Three scan modes of full, SIM and SRM were recorded. The time period and scanned m/z ranges of each segment, vitamin molecular weights, precursor ions, collision energies and fragment ions used for quantifications are reported in Table 4.

## Figures and Tables

**Table 1:** Calibration data, limits of detection (LOD), limits of quantification (LOQ) and range of linearity.

	Tears					Serum				
Water-soluble vitamin	Calibration curves	Correlation	LOD (ng)	LOQ (ng)	Linear range (μM)	Calibration curves	Correlation	LOD (ng)	LOQ (ng)	Linear range (μM)
B_1_, Thiamine	y = 1.2777x + 0.001	0.9966	0.075	0.25	0.01–50	y = 0.6353x + 0.002	0.9993	0.061	0.20	0.008–50
B_2_, Riboflavin	y = 0.4048x + 0.002	0.9990	0.12	0.40	0.01–100	y = 0.3937x + 0.004	0.9979	0.077	0.26	0.008–100
B_3_, Nicotinamide	y = 0.1008x + 0.006	0.9976	0.78	2.6	0.3–100	y = 0.1178x + 0.006	0.9980	0.57	1.9	0.2–100
B_5_, Pantothenic acid	y = 0.0054x + 0.000	0.9977	0.93	3.1	0.3–200	y = 0.0054x + 0.000	0.9977	0.76	2.5	0.1–200
B_9_, Folic acid	y = 0.0454x + 0.008	0.9964	0.30	1.0	0.03–10	y = 0.0457x + 0.008	0.9964	0.36	1.2	0.03–10
Fat-soluble vitamin	Calibration curves	Correlation	LOD (ng)	LOQ (ng)	Linear range (μM)	Calibration curves	Correlation	LOD (ng)	LOQ (ng)	Linear range (μM)
A, Retinol						y = 0.1837x + 0.004	0.9947	1.4	4.6	0.2–20
E, Tocopherol	y = 1.0133x + 0.005	0.9982	0.18	0.58	0.02–20	y = 0.5276x + 0.003	0.9958	0.42	1.4	0.04–100

**Table 2:** Recovery, intra-day (n=6) and inter-day (n=7) precision for detection of water-soluble and fat-soluble vitamins under two LC-MS/MS methods.

Sample	Vitamin	added [uM]	Intra-day (n=6)			Inter-day (n=7)		
			Found [uM]	RSD [%]	Recovery [%]	Found [uM]	RSD [%]	Recovery [%]
Tear	B1	0.50	0.48 ± 0.03	5.9	95.8	0.52 ± 0.03	5.6	108
						
		1.0	1.1 ± 0.1	6.7	109	0.97 ± 0.09	9.4	97.2
		1.5	1.4 ± 0.0	3.0	91.0	1.5 ± 0.08	5.8	96.8
							
	B2	0.50	0.50 ± 0.04	8.3	99.7	0.54 ± 0.06	10	107
		1.0	0.87 ± 0.07	8.2	87.2	0.92 ± 0.07	7.7	92.0
		1.5	1.4 ± 0.1	8.6	92.6	1.4 ± 0.06	4.4	96.2
							
	B3	0.50	0.52 ± 0.03	6.6	103	0.49 ± 0.04	9.1	98.5
		1.0	0.92 ± 0.03	3.7	91.7	0.98 ± 0.06	5.7	97.7
		1.5	1.4 ± 0.0	2.5	92.1	1.5 ± 0.07	4.8	101
							
	E	0.50	0.48 ± 0.02	4.3	95.8	0.45 ± 0.04	9.7	89.1
		1.0	0.99 ± 0.04	3.7	99.0	0.98 ± 0.07	6.8	98.5
		1.5	1.3 ± 0.1	5.8	87.2	1.3 ± 0.12	9.8	84.8
								
Serum	B1	0.50	0.45 ± 0.02	3.4	90.1	0.46 ± 0.03	5.9	93.9
		1.0	0.91 ± 0.06	6.5	91.2	0.96 ± 0.06	6.2	91.6
		1.5	1.4 ± 0.0	6.5	93.2	1.4 ± 0.1	5.2	95.0
							
	B2	0.50	0.50 ± 0.03	6.6	99.9	0.48 ± 0.04	8.9	98.7
		1.0	0.97 ± 0.03	3.4	96.9	1.0 ± 0.1	6.5	97.6
		1.5	1.4 ± 0.1	4.0	93.1	1.5 ± 0.1	5.5	98.2
							
	B3	0.50	0.50 ± 0.04	8.7	99.7	0.54 ± 0.04	7.5	98.8
		1.0	1.0 ± 0.1	7.5	100	0.95 ± 0.06	6.5	96.4
		1.5	1.5 ± 0.0	2.2	102	1.5 ± 0.1	4.9	98.6
							
	B5	0.50	0.48 ± 0.07	15	95.4	0.46 ± 0.05	12	91.8
		1.0	1.0 ± 0.2	18	101	0.93 ± 0.15	16	90.6
		1.5	1.3 ± 0.2	12	84.8	1.4 ± 0.2	15	91.7
							
	B9	0.50	0.48 ± 0.03	7.0	95.8	0.46 ± 0.03	6.0	87.5
		1.0	0.97 ± 0.14	14	96.9	0.94 ± 0.14	14	98.2
		1.5	1.5 ± 0.1	8.7	100	1.5 ± 0.1	5.6	103
							
	A	1.0	0.93 ± 0.08	8.1	93.1	0.95 ± 0.04	3.9	95.1
		2.0	2.0 ± 0.2	7.9	99.2	1.9 ± 0.2	8.2	95.6
		3.0	2.8 ± 0.2	8.6	93.8	2.8 ± 0.2	8.8	93.1
							
	E	10	8.4 ± 0.2	2.8	84.0	8.9 ± 0.8	8.6	88.6
		20	18 ± 1	8.2	88.2	17 ± 1	3.6	85.0
		30	26 ± 2	6.1	87.3	26 ± 1	4.5	86.0

**Table 3:** Chromatography and mass spectrometry parameters for detection of water-soluble vitamins.

Time period (min)	Scanned range (m/z)	Vitamins	Molecular weight (Da)	Precursor ion (m/z)	Collision energy (eV)	Fragment ion for quantification (m/z)
0-3.5	200-300	B_1_, Thiamine	265	265 [M]^+^	20	122
				
		Thiamine-[^13^C_4_]	269	269 [M]^+^	20	122
						
3.5–10	100–200	B_3_, Nicotinamide	122	123 [M+H]^+^	0	123
		Nicotinamide-[D_4_]	126	127 [M+H]^+^	0	127
		B_6_, Pyridoxine	169	170 [M+H]^+^	18	152 [M+H −H2O]^+^
		Pyridoxine-[D_3_]	172	173 [M+H]+	18	155 [M+H −H2O]^+^
						
10–18	200–700	B_2_, Riboflavin	376	377 [M+H]^+^	23	243
		Riboflavin-[^13^C_4_,^15^N_2_]	382	383 [M+H]^+^	23	249
		B_5_, Pantothenic acid	219	220 [M+H]^+^	18	90
		Pantothenate-[^13^C_3_,^15^N]	223	224 [M+H]+	18	94
		B_7_, Biotin	244	245 [M+H]^+^	16	227 [M+H −H_2_O]^+^
		Biotin-[D_2_]	246	247 [M+H]^+^	16	229 [M+H −H_2_O]^+^
		B_9_, Folic acid	441	442 [M+H]^+^	19	295
		B_12_, Cynaocobalamin	1355	678 [M/2+H]^+^	17	359

**Table 4:** Chromatography and mass spectrometry parameters for detection of fat-soluble vitamins.

Time period (min)	Scanned range (m/z)	Vitamins	Molecular weight (Da)	Precursor ion (m/z)	Collision energy (eV)	Fragment ion for quantification (m/z)
0-10	200-450	A, Retinol	286	269 [M+H-H_2_O]^+^	25	213
				
		Retinol-[D_5_]	291	274 [M+H-H_2_O]^+^	25	218
		25(OH)D_3_	400	401 [M+H]^+^	16	383 [M+H −H_2_O]^+^
						
10–18	300–500	D_3_, Choleocalciferol	384	385 [M+H]^+^	22	367 [M+H −H_2_O]^+^
		E, α-Tocopherol	430	429 [M+H-H2]^+^	27	165
		Tocopherol-[D_6_]	436	435 [M+H-H_2_]^+^	27	171
						
18–25	400–500	K_1_, Phylloquinone	450	451 [M+H]^+^	25	187
		Phylloquinone-[D_7_]	457	458 [M+H]^+^	25	194
