# Supplementary material for: Data representing two separate LC-MS methods for detection and quantification of water-soluble and fat-soluble vitamins in tears and blood serum
Source: Data Brief. 2017 Feb 16;11:316–30. doi: 10.1016/j.dib.2017.02.033 (PMC5328915; doi:10.1016/j.dib.2017.02.033)
Supplement: Supplementary file 1 — Supplementary material [file mmc1.docx]

Instructions say:

Conflict of Interest is mandatory for revision, so while submitting please submit the file by selectin the description from the drop down. Please click here to download the Conflict of Interest form.

The ‘click here’ isn’t hyperlinked to a form, so please send it to [Minerick@mtu.edu](mailto:Minerick@mtu.edu) for completion.

The authors declare no conflict of interest.
